# Novel N,N-dialkyl cyanocinnamic acids as monocarboxylate transporter 1 and 4 inhibitors

**DOI:** 10.18632/oncotarget.26760

**Published:** 2019-03-22

**Authors:** Shirisha Jonnalagadda, Sravan K. Jonnalagadda, Conor T. Ronayne, Grady L. Nelson, Lucas N. Solano, Jon Rumbley, Jon Holy, Venkatram R. Mereddy, Lester R. Drewes

**Affiliations:** ^1^ Integrated Biosciences Graduate Program, University of Minnesota, Duluth, MN 55812, USA; ^2^ Department of Pharmacy Practice & Pharmaceutical Sciences, University of Minnesota, Duluth, MN 55812, USA; ^3^ Department of Biomedical Sciences, Medical School Duluth, University of Minnesota, Duluth, MN 55812, USA; ^4^ Department of Chemistry and Biochemistry, University of Minnesota, Duluth, MN 55812, USA

**Keywords:** monocarboxylate transporter 1 inhibitor, monocarboxylate transporter 4 inhibitor, 2-alkoxy-*N,N*-dialkyl cyanocinnamic acid, cancer, metabolism

## Abstract

Potent and dual monocarboxylate transporter (MCT) 1 and 4 inhibitors have been developed for the first time as potential anticancer agents based on α-cyanocinnamic acid structural template. Candidate inhibitors 1–9 have been evaluated for *in vitro* cell proliferation against MCT1 and MCT4 expressing cancer cell lines. Potential MCT1 and MCT4 binding interactions of the lead compound 9 have been studied through homology modeling and molecular docking prediction. *In vitro* effects on extracellular flux via glycolysis and mitochondrial stress tests suggest that candidate compounds 3 and 9 disrupt glycolysis and OxPhos efficiently in MCT1 expressing colorectal adenocarcinoma WiDr and MCT4 expressing triple negative breast cancer MDA-MB-231 cells. Fluorescence microscopy analyses in these cells also indicate that compound 9 is internalized and concentrated near mitochondria. *In vivo* tumor growth inhibition studies in WiDr and MDA-MB-231 xenograft tumor models in mice indicate that the candidate compound 9 exhibits a significant single agent activity.

## INTRODUCTION

Metabolic reprogramming is now recognized as a critical hallmark of cancer and by understanding and manipulating the energetics of tumor metabolism, new therapeutic strategies may be developed for the treatment of cancer [1–8]. The survival and progression of tumors is accompanied by a significant increase in the metabolic enzymes and transporters, along with the cooperative reprogramming of other cells in the stromal compartment including cancer associated fibroblasts that assist tumor growth [9–13].

Glycolysis is generally amplified in cancer cells to keep up with bioenergetic and biosynthetic demands for rapid cell proliferation [14–17]. Anabolic and proliferative cancer cells also utilize the catabolic by-products of glycolysis such as lactate and pyruvate to fuel TCA cycle and mitochondrial OxPhos for further ATP generation to meet synthetic and energetic needs [14–17]. These metabolic transformations that support tumor progression result in overexpression of numerous enzymes and transporters, hence, provide an opportunity for pharmacological intervention [18, 19]. Several studies also recognize the importance of mitochondrial OxPhos to generate a large portion of ATP in cancer cells [20–22]. OxPhos also plays an important role in cancer cell survival, drug resistance, relapse, and metastasis. OxPhos intermediates are utilized in the TCA cycle and many are shuttled into numerous biosynthetic pathways including fatty acids, amino acids, and nucleotides. In this regard, inhibition of OxPhos will lead to severe ATP depletion and dysfunction of the TCA cycle, again starving cancer cells of critical components for cell survival and proliferation [20–22].

Monocarboxylic acid transport is one of the metabolic targets wherein the flux of small ketone bodies such as lactic acid and pyruvic acid occurs to support metabolic demands in cancer cells [23–29]. Monocarboxylic acid transporters (MCTs) are members of the solute carrier family 16 (SCL16 family) and consist of 14 known isoforms. Of these, only MCTs 1–4 have been shown to catalyze the bidirectional proton-linked transport of monocarboxylates such as lactate, pyruvate, and some ketone bodies. MCTs are present in the cell membrane and are centrally involved in glycolysis to efflux the end product lactate out of the tumor cells to avoid the decrease in intracellular pH which may lead to apoptosis [23–29]. MCT1 and MCT4 are encoded by the genes SCL16A1 and SLC16A3 and they also play an active role in the shuttling of lactate from glycolytic cancer cells into the neighboring oxidative cells for energy generation via mitochondrial OxPhos [9–13]. Hence, MCT1 and MCT4 are important therapeutic targets for metabolism-directed cancer treatments [30–37].

## RESULTS

### 2-Methoxy-4-N,N-dialkyl cyanocinnamic acids are dual MCT1 and MCT4 inhibitors

**Figure 1:**
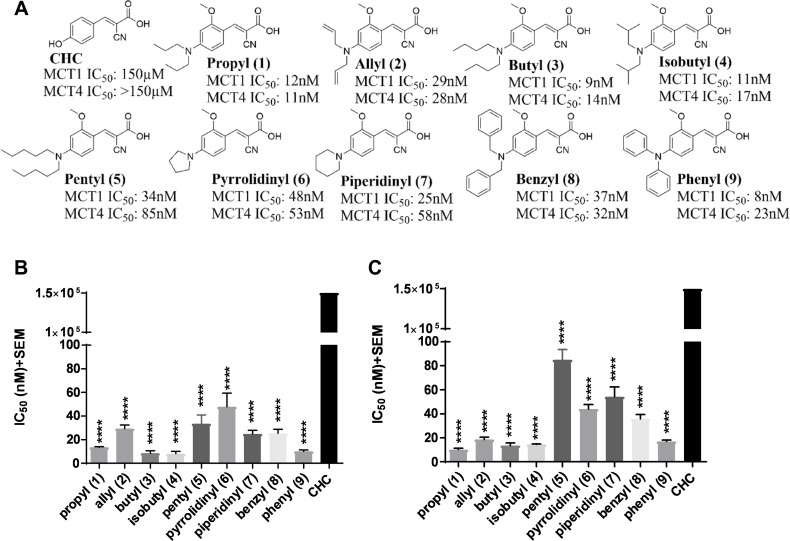
MCT1 and MCT4 lactate uptake inhibition (**A**) Chemical structures of 2-methoxy-4-*N,N*-dialkyl cyanocinnamic acids 1–9. Bar graphs of (**B**) MCT1 inhibition and (**C**) MCT4 inhibition using lactate uptake study with compounds 1–9 in comparison to CHC. The final average ± sem of at least three independent experimental values were calculated. Repeated measures one-way ANOVA was used to calculate statistical significance (*P* < 0.05) between test compounds and CHC. ^****^P < 0001.

Several recent studies have reported the importance of MCT1 and MCT4 in various cancers [23–37]. These studies indicate that elevated expression of MCT1 and/or MCT4 is correlated with poor patient prognosis and increased patient mortality in cancer patients [23–37]. Therefore, targeting MCT1 and/or MCT4 is of high therapeutic importance. In this regard, our previous structure activity relationship studies using CHC (Figure 1A) template indicated that placing *N,N-*dialkyl/diaryl groups at the 4-position and a methoxy (-OMe) group at the 2-position proved to be the most optimized structural moiety for MCT1 inhibition [32, 33]. L-[^14^C]-lactate uptake studies on MCT1 expressing rat brain endothelial-4 (RBE4) cells revealed several 2*-*methoxy-4*-N*,*N*-dialkyl cyanocinnamic acids 1–9 as potent inhibitors of MCT1 at low nanomolar concentrations in our earlier study (Figure 1B) [32, 33].

Because compounds 1–9 exhibited potent MCT1 inhibition, we investigated if these candidates would also inhibit the MCT4 function. For this purpose, a triple negative breast cancer (TNBC) cell line MDA-MB-231 was utilized. These cells predominantly express MCT4 as confirmed by Western blot and quantitative PCR analysis (Supplementary Figure 1). Upon evaluation of compounds 1–9 using L-[^14^C]-lactate uptake study, they were also found to exhibit excellent inhibitory activity against MCT4 (Figure 1C, Supplementary Table 1). Compared to CHC (IC_50_ ≥ 150 μM), compounds 1–9 exhibited several thousand-fold greater potency in inhibiting MCT1 (IC_50_ 8–48 nM) [32, 33] and MCT4 (IC_50_ 11–85 nM). Furthermore, compounds 1–9 were equally potent against both MCT1 and MCT4. These results constitute the first report of dual inhibition of MCT1 and MCT4 in nanomolar potency using small molecules.

### MCT1 and MCT4 inhibitors do not affect cell proliferation in MDA-MB-231 and WiDr cells

Encouraged by dual inhibition of MCT1/4, we then evaluated cell proliferation of compounds 1–9 using SRB assay. MCT1 expressing cells WiDr and MCT4 expressing cells MDA-MB-231 were chosen for this assay (Supplementary Figure 1A). Compounds 1–8 did not show any appreciable cell proliferation inhibition up to 25 μM in both MDA-MB-231 and WiDr cell lines. Although compound 9 exhibited an IC_50_ of 4.2 μM in WiDr cell line, it did not show any activity in MDA-MB-231 cell line (Table 1).

**Table 1:** SRB IC_50_^*^ (μM) values of 2-methoxy *N,N*-dialkyl cyanocinnamates in MDA-MB-231 and WiDr cell lines

Compound	MDA-MB-231	WiDr
Propyl (1)	>25	>25
Allyl (2)	>25	>25
Butyl (3)	>25	>25
Isobutyl (4)	>25	>25
Pyrrolidinyl (6)	>25	>25
Piperidinyl (7)	>25	>25
Benzyl (8)	>25	>25
Phenyl (9)	>25	4.2 ± 0.4

^*^The experiments were carried out in duplicate wells and the average ± sem values of minimum three separate experiments was calculated.

### Glycolysis stress test of compound 9 result in potent inhibition of glycolysis

To evaluate the metabolic profile of these MCT1 and MCT4 inhibitors, extracellular flux using Seahorse XFe96^®^ assay was performed. Based on the lipophilic structural features and enhanced cell proliferation inhibition properties of these compounds, it is quite possible that they interact with intracellular organelles, including the mitochondria. In this regard, we chose candidate compound 9 based on its potent MCT1 and MCT4 inhibition and previously demonstrated ability to reduce tumor growth in a WiDr mouse xenograft model [32, 33]. To further investigate the metabolic implications of MCT1 and MCT4 inhibition, we also compared compound 9 with AZD3965 and CHC. AZD3965 is a known MCT1 inhibitor with no significant MCT4 inhibition activity [36, 37] For these studies, we utilized WiDr and MDA-MB-231 cells.

**Figure 2:**
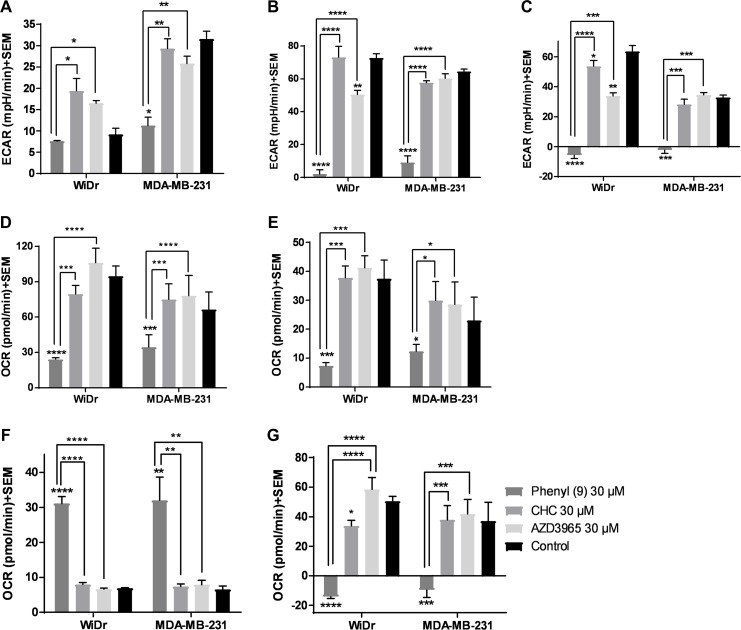
Glycolysis and mitochondrial stress tests of compound 9, CHC, and AZD3965 (**A–C**) represent the parameters from glycolysis stress test: (**A**) glycolysis, (**B**) glycolytic capacity, and (**C**) glycolytic reserve of compounds at 30 μM concentration in MCT1 expressing WiDr and MCT4 expressing MDA-MB-231 cells. (**D–G**) represent the parameters from mitochondrial stress test: (**D**) maximal respiration, (**E**) ATP production, (**F**) proton leak, and (**G**) spare respiratory capacity in WiDr and MDA-MB-231 cells. The ECAR and OCR values of were calculated using wave software. The average + SEM values of at least three independent experimental values were calculated. ^*^P < 0.05, ^**^P < 0.01, ^***^P < 0.001, ^****^P < 0.0001.

In the GST, three parameters namely glycolysis, glycolytic capacity and glycolytic reserve were measured. Our results show that compound 9 decreased glycolytic capacity in MCT1 expressing WiDr and MCT4 expressing MDA-MB-231 cells at 30 μM, whereas, AZD3965 decreased glycolytic capacity only in WiDr at 30 μM (Figure 2A–2C, Supplementary Figure 2A–2B). A similar trend was observed in glycolytic reserve for candidate 9 in both cell lines, and it completely arrested glycolytic reserve implying energy is not generated via glycolysis or other proton producing metabolic pathways. CHC and AZD3965 decreased glycolytic reserve only in MCT1 expressing WiDr. It is interesting to note that compound 9 showed significantly greater inhibition of glycolytic parameters compared to AZD3965 and CHC in WiDr and MDA-MB-231 cells (Figure 2A–2C). AZD3965 did not exhibit significant difference in the inhibition of glycolytic parameters compared to CHC.

### Mitochondrial stress test of compound 9 result in significant inhibition of mitochondrial parameters

The MST results indicated that compound 9 significantly decreased maximal respiration, ATP production and spare respiratory capacity in WiDr and MDA-MB-231 cells as observed by the decrease in OCR (Figure 2D–2G, Supplementary Figure 2C–2D). CHC and AZD3695 did not affect these parameters in the two cell lines implying these compounds don't inhibit or effect mitochondrial OxPhos. While candidate compound 9 significantly increased proton leak in both the cell lines, CHC and AZD3965 did not affect proton leak in either cells (Figure 2F), indicating that a significant portion of 9 is also internalized into the cytoplasm, causing disruption of mitochondrial function.

### Compounds 2 and 9 result in significant inhibition of glycolytic and mitochondrial parameters

**Figure 3:**
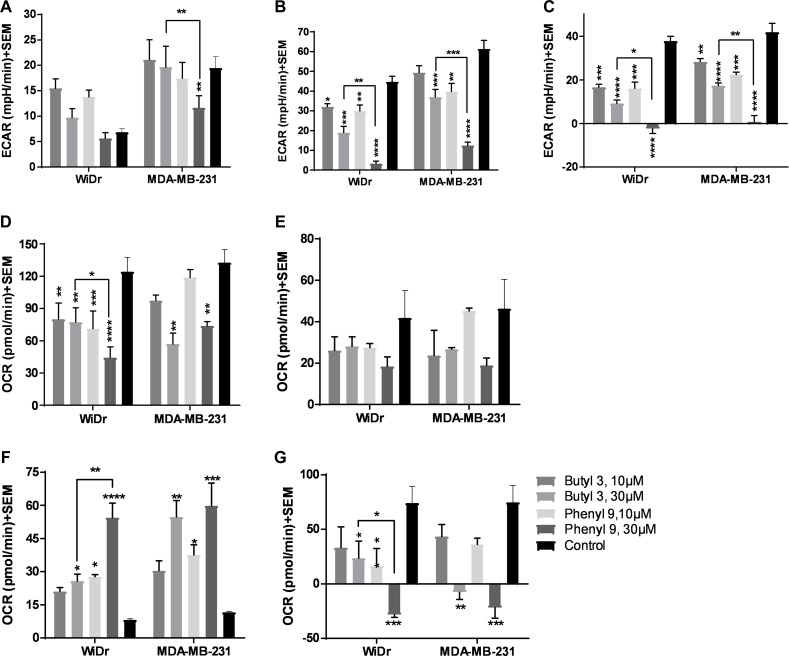
Glycolysis and mitochondrial stress tests of compounds 3 and 9 (**A–C**) represent the parameters from glycolysis stress test: (**A**) glycolysis, (**B**) glycolytic capacity, and (**C**) glycolytic reserve of compounds at 30 μM concentration in WiDr and MDA-MB-231 cells. (**D–G**) represent the parameters from mitochondrial stress test: (**D**) maximal respiration, (**E**) ATP production, (**F**) proton leak, and (**G**) spare respiratory capacity in WiDr and MDA-MB-231 cells. The ECAR and OCR values of were calculated using wave software. The average + SEM values of at least three independent experimental values were calculated. ^*^P < 0.05, ^**^P < 0.01, ^***^P < 0.001, ^****^P < 0.0001.

To further explore the potential of compounds in crossing the cell membrane and effecting cellular metabolic properties we also investigated compound 3 and compared it to compound 9. Compound 3 has two butyl groups and one phenyl ring, whereas compound 9 has three phenyl rings, and both compounds are equipotent in terms of MCT1 and MCT4 inhibition. In GST, both butyl 3 and phenyl 9 showed a significant decrease in glycolytic capacity and glycolytic reserve compared to control at 30 μM in WiDr (Figure 3A–3C, Supplementary Figure 3A–3B). Interestingly, compound 9 exhibited a significant difference in the above-mentioned glycolysis parameters compared to 3, making it superior to the compound 3. Similar glycolytic inhibition trends were also observed in MDA-MB-231 with compound 3 (Figure 3A–3C). In this case also candidate 9 was found to be superior compared to 3 in disrupting glycolysis. For MST, while 3 and 9 decreased maximal respiration, ATP production, and spare respiratory capacity, compound 9 exhibited superior inhibition properties over compound 3 in the above-studied parameters (Figure 3D–3G, Supplementary Figure 3C–3D). A similar profile was observed for proton leak in which candidate 9 significantly increased proton leak compared to compound 3 in WiDr (Figure 3F).

### MitoTracker staining indicates that compound 9 localizes in areas near mitochondria

**Figure 4:**
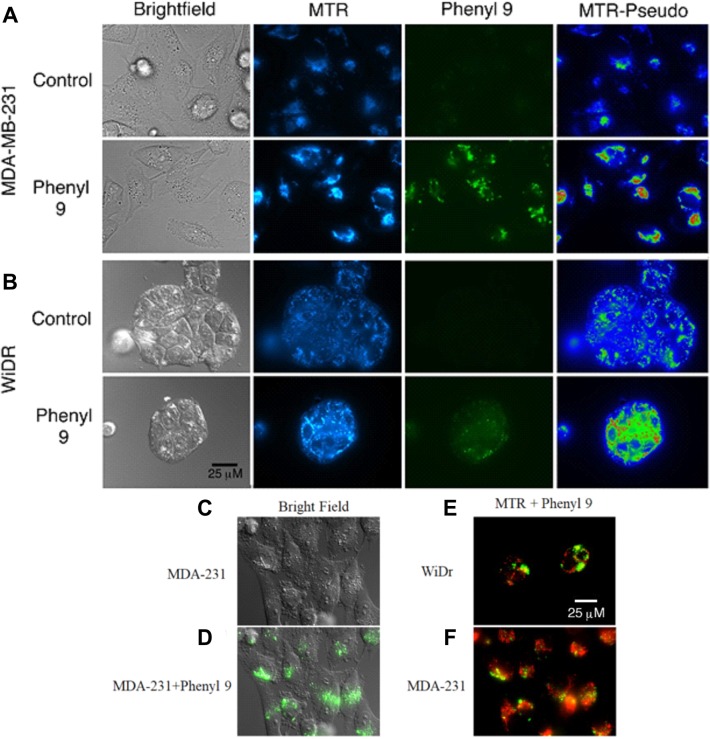
Mitotracker red staining in compound 9 treated MDA-MB-231 and WiDr cell lines Representative pictures of (**A**) MDA-MB-231 and (**B**) WiDr cells after exposure to compound 9 (green) for 1 hour and MitoTracker red (MTR) for 15 minutes. Compound 9 is localized in regions of higher mitochondrial density in WiDr cell line. MTR-Pseudo images show the MTR signal pseudocolored using the Rainbow RGB LUT of the FIJI software program, to demonstrate mitochondrial hyperpolarization after addition of compound 9. (**C, D**) Compound 9 localizes to granular regions of MDA-MB-231 cells. Compound 9 localizes to regions near to, but does not overlap with, most mitochondria (red) in both (**E**) WiDr and (**F**) MDA-MB-231 cells. Images are representative of multiple fields of view from three independent experiments. Scale bar, 25 μm.

Our studies showed that compound 9 is fluorescent (470/40 excitation, 525/50 barrier filters) and can be imaged with a fluorescein or GFP filter set (Supplementary Figures 4 and 5). To investigate cellular uptake and localization of compound 9, we have carried out fluorescence microscopy studies in WiDr and MDA-MB-231 cells, along with MitoTracker red to test for mitochondrial perturbation. Interestingly, it was observed that compound 9 was internalized in both cell lines (Figure 4A and 4B). In MDA-MB-231 cells, compound 9 localized to granular regions of cytoplasm (Figure 4C and 4D). In both cell lines, compound 9 was concentrated in areas near mitochondria, but did not appear to co-localize with most mitochondria (Figure 4E and 4F).

### Homology modeling of and computational inhibitor docking to human MCT1 and MCT4 indicate that the phenyl rings in compound 9 are involved in hydrophobic interactions

**Figure 5:**
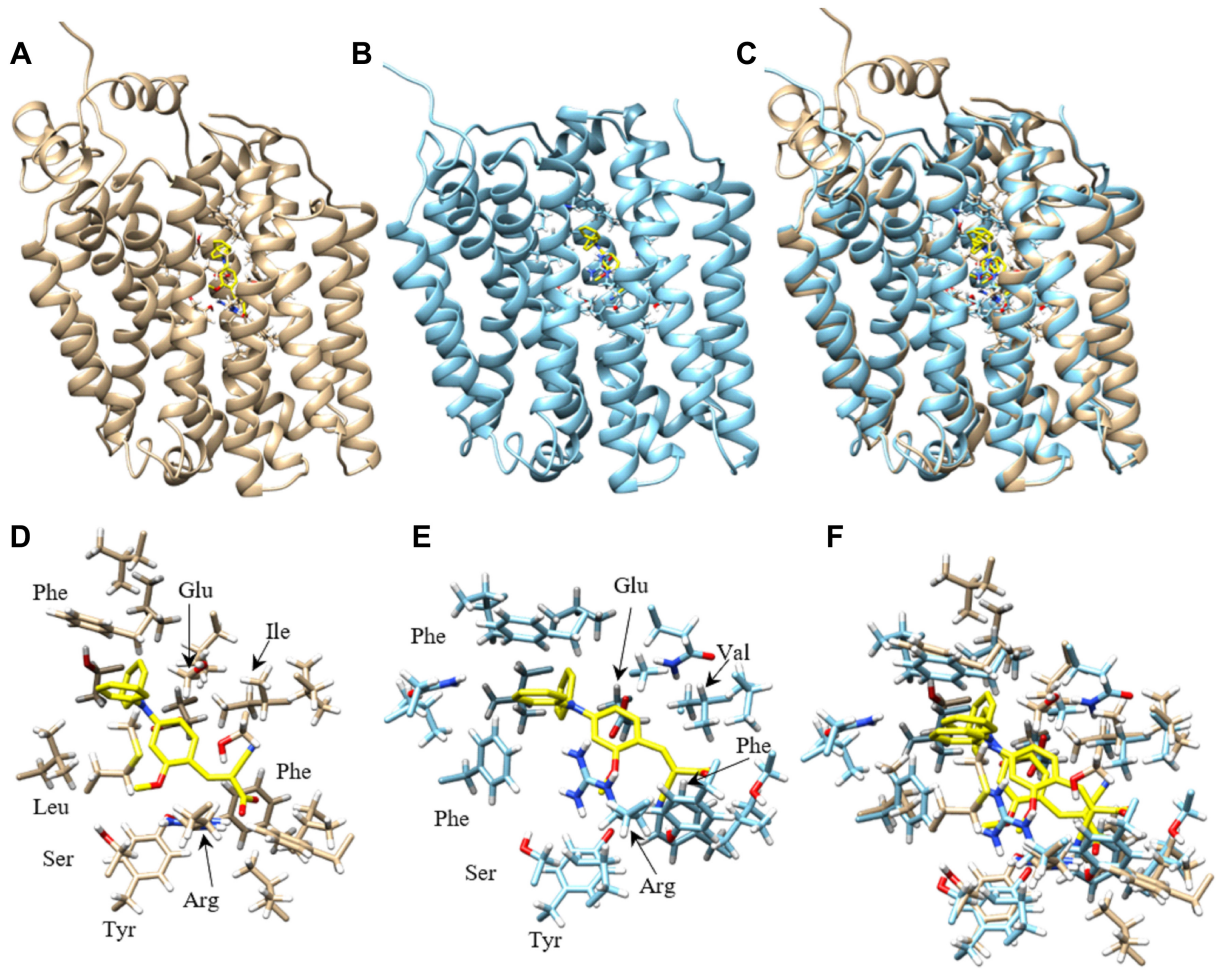
Homology model of human MCT1 and MCT4 docked with compound 9 Most favorable compound 9 binding pose to human MCT1 and MCT4 were represented. (**A**) Cα ribbon homology structure of MCT1 with docked compound 9 (yellow) and binding site residues within 4.5 Å shown. (**B**) Cα ribbon homology structure of MCT4 with docked compound 9 (yellow) and binding site residues within 4.5 Å shown. (**C**) Overlay of MCT1 and MCT4 homology models and their respective best compound 9 docking pose. (**D**) Compound 9 (yellow) and residue forming its binding site in MCT1, all residues within 4.5 Å are shown. (**E**) Compound 9 (yellow) and residue forming its binding site in MCT1, all residues within 4.5 Å are shown. (**F**) Overlay of most favorable binding pose of compound 9 for MCT1 and MCT4 and all residues within 4.5 Å. Models were displayed with Chimera.

To understand the potential molecular interactions of MCT1 and MCT4 inhibitors, homology modeling and computational docking studies were performed. Optimal homology models were selected primarily based on an evaluation of charged residue rotamer orientation in the transmembrane spans. The resulting human MCT1 structure was compared to a previously reported rat MCT1 homology model based on an *E. coli* glycerol-3-phosphate transporter template [38]. For comparison, we analyzed the residues involved in inhibitor binding between our human MCT1 structure and compound 9. In order to achieve an unbiased ligand/inhibitor binding pocket search, our inspection area included the entire transmembrane spanning domain and extended into the inward-open aqueous surface of MCT1 and MCT4. The best ranked docking pose of compound 9 to both MCT1 and MCT4 was determined to be nearly structurally indistinguishable (Figure 5). Compound 9 is surrounded by a number of aliphatic and aromatic side chains. The binding affinity of compound 9 was estimated to be –9.2 kcal/mol for MCT1 and –9.6 kcal/mol for MCT4, consistent with the compounds high affinity for both proteins determined experimentally. The estimated binding affinity of parent compound CHC for MCT1 was –6.4 kcal/mol, an approximately 220-fold lower affinity. Further, of the top 18 binding poses determined for compound 9 binding to MCT1, 13 of 18 occupied the same binding site while 6 of 18 poses occupied the analogous MCT4 site. Only 2 of 18 poses for parent compound CHC binding to MCT1 were structurally similar, a surrogate for binding specificity.

Nancolas et al. determined the best binding pose of AstraZeneca MCT1 inhibitor AR-C155858 to the homology model of rat MCT1 [39]. A small list of amino acids determined to form hydrogen bonds with inhibitor were identified. Although our inhibitor is quite structurally distinct from AR-C155858, the residues contacting inhibitor in our study were highly analogous or structurally very near the rat MCT1 residues. Analogous amino acids included Tyr34, Arg306, Ser364, Leu367 and Glu391 in rat MCT1 and Tyr34, Arg313, Ser371, Leu374 and Glu398 identified in the human MCT1/compound 9 complex (Supplementary Table 2).

### Compound 3 reduces the tumor burden in MCT1 expressing WiDr xenograft model

**Figure 6:**
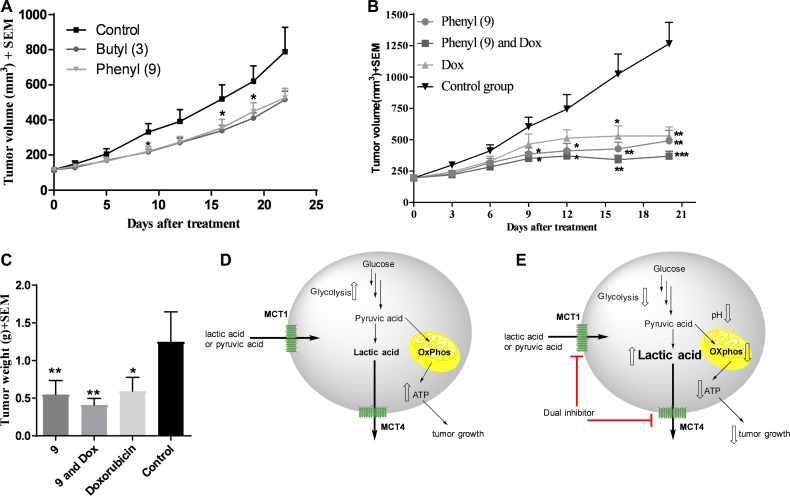
*In vivo* xenograft studies in WiDr and MDA-MB-231 tumor models (**A**) WiDr tumor xenograft study of compound 3 and compound 9. Mice (*n* = 8) were treated with 8 mg/kg of compound 3, intraperitoneally, two times a day. (**B**) Tumor growth inhibition study with compound 9 in MDA-MB-231 tumor xenograft model (*n* = 6). Mice were treated with compound 9 (70 mg/kg, ip, bid until day-4; qd from day-5), a combination of 9 and doxorubicin (0.5 mg/kg, ip, five days a week), and doxorubicin. (**C**) Tumor growth inhibition based on isolated tumor mass. ^*^P < 0.05, ^**^P < 0.01, ^***^P < 0.001, ^****^P < 0.0001. Schematic representation of (**D**) untreated tumor cells and (**E**) inhibition of MCT1 and MCT4 and decreased glycolysis and mitochondrial OxPhos in compound 9 treated tumor cells. Upward hollowed arrow indicates “increase” in function/amount and downward hollowed arrow indicates “decrease” in function/amount.

Our earlier studies indicated that candidate compound 9 exhibited significant tumor growth inhibition in WiDr tumor model [32, 33]. Although compound 3 exhibits inferior effects on glycolytic and mitochondrial properties compared to 9, we investigated its anticancer efficacy in a WiDr tumor model for *in vivo* comparison with compound 9. The butyl derivative 3 exhibited similar tumor growth inhibition to that of compound 9 (Figure 6A).

### Compound 9 not only inhibits tumor growth in WiDr, but also in MCT4 expressing MDA-MB-231 tumor model

Based on good tumor growth inhibition with compound 9 in WiDr tumor model, and also based on its superior metabolic disruption properties compared to 3, compound 9 was further advanced for *in vivo* studies in the MCT4 expressing MDA-MB-231 tumor xenograft model. Group-1 was administered with compound 9, group-2 was given a combination of compound 9 and clinical breast cancer drug doxorubicin (AKSci catalog # E518), and group-3 was treated with doxorubicin alone. Group-4 was assigned as a control group and treated with vehicle (10% DMSO in saline). The treatment was continued up to 18 days and on day 20, the mice were euthanized and tumor masses isolated and weighed. Tumor growth inhibitions were found to be 58, 67 & 48% in groups 1, 2 and 3, respectively based on tumor volume (Figure 6B) and 56, 67 & 52% in groups 1, 2 and 3, respectively based on isolated tumor weights (Figure 6C). These studies clearly exhibit the potential of MCT1/4 inhibitors in TNBC treatment. Our *in vivo* pharmacokinetic studies also indicated that peak plasma concentration was observed at 15 minutes and most of the compound was eliminated in less than one hour [32]. Due to these reasons, higher dosages of compound 9 were required to produce significant anticancer efficacy *in vivo*. In all these studies, <20% of body weight loss was observed.

## DISCUSSION

MCT1 and MCT4 are upregulated in various cancers and the presence of either of these markers is linked with poor patient prognosis [23–37]. MCT1 and MCT4 are frequently credited with lactate transport in and out of cells, respectively. However, shuttling of lactate via MCTs is bidirectional and dependent on the pH and anion gradients [40]. Hence, low intracellular pH favors lactate efflux and high intracellular pH favors lactate influx.

Based on their excellent MCT1 inhibition activity in low nanomolar potency [32, 33], the candidate compounds 1–9 were evaluated for MCT4 inhibition. All compounds exhibited similar inhibitory profile for both MCT1 and MCT4 with slight preference for MCT1 over MCT4 (Figure 1B). Based on these results, the mode of action of compounds appears to be similar for MCT1 and MCT4. In this regard, potential MCT1 and MCT4 binding interactions have been studied through homology modeling and molecular docking prediction (Figure 5).

The structures of inward-open human MCT1 and MCT4 generated here appear to be of sufficient quality to identify the binding site and reason for dual specificity of compound 9. The binding site amino acids for compound 9 in MCT1 and MCT4 were predicted to be identical based on model. The concordance of binding site residues for AstraZeneca AR-C155858 inhibitor binding to a rat MCT1 model, although not fully expected for such a structurally distinct inhibitor, lends confidence in the results obtained here. The lipophilic phenyl groups of compound 9 binding to MCT1 and MCT4 is characterized by a number of hydrophobic contacts, including aromatic stacking to phenylalanine in both proteins. The extensive hydrophobic contact surface likely leads to a dramatic increase in affinity over CHC, supplemented by several putative hydrogen bonds. All polar atoms in compound 9 are immediately adjacent to one or more polar side chains, including conserved Tyr34, Ser154/156 and Arg313/278 (Figure 5). The 2-methoxy group specifically interacts with Tyr34, another strong contributor to specificity and high affinity over CHC. Of the residues within 4.5Å of compound 9 the most obvious unsatisfied interaction is that of conserved Glu398/363, also identified in the binding site of AstraZeneca inhibitor AR-C155858 in rat MCT1 [39].

Since compounds 1–9 exhibited potent dual MCT1 and MCT4 inhibition, we then evaluated cell proliferation studies of these compounds in cancer cell lines. SRB assay results indicate that compound 9 significantly inhibits cell proliferation of WiDr cells (Table 1). This is not surprising as it is known that potent inhibition of MCT may not lead to corresponding levels of cell proliferation inhibition [30]. *In vitro*, cells are exposed to supraphysiological levels of oxygen, nutrients, and growth factors which may render them more resistant to some types of metabolic perturbation. Also, the tumor microenvironment *in vivo* can be expected to include more drug targets than a single cultured cell line, due to the presence of potentially metabolically-coupled stromal cells, and other cell signaling effects.

In GST, MDA-MB-231 produced high ECAR indicating that these cells pursue glycolysis as a dominant energy source, whereas WiDr are less glycolytic in nature compared to MDA-MB-231 as evidenced by the low ECAR in the control wells in the presence of glucose (Figure 2A). In MDA-MB-231 and WiDr cells, 9 lead to a significant disruption in glycolytic capacity and glycolytic reserve. CHC, being a weak MCT1 and MCT4 inhibitor, did not affect glycolysis and glycolytic capacity in both the cell lines. Being a selective MCT1 inhibitor, AZD3965 decreased glycolytic capacity and glycolytic reserve only in WiDr. However, candidate 9 was found to be superior to AZD3965 in inhibiting glycolytic parameters. AZD3965 did not show any glycolysis inhibition in MCT4 expressing MDA-MB-231.

We then investigated if 9 would disrupt mitochondrial OxPhos. Our results from MST suggest that 9 crosses the plasma membrane and effects mitochondria by causing an increase in proton leak and inhibiting ATP production (Figure 2E, 2F). Treatment with 9 was found to prevent the cells from meeting their energy demands by not only decreasing glycolytic reserve (Figure 2C), but also efficiently suppressing spare respiratory capacity (Figure 2G) leading to an even greater energy crisis in both GST and MST. These results suggest that compound 9 has pleiotropic activities effecting glycolysis and mitochondrial OxPhos. In this study, CHC at 30 μM resulted in a very limited effect on mitochondria, and AZD3965 did not affect any mitochondrial parameters indicating that this compound is more selective towards plasma membrane MCT1 inhibition.

We also investigated the efficacy of another MCT inhibitor 3 on glycolysis and mitochondrial parameters and compared it to 9. Although compound 3 exhibited significant inhibition of GST and MST parameters (Figure 3A–3G), compound 9 was still found to exhibit superior efficacy compared to 3. CHC and other related cyanocinnamic acid derivatives have been previously reported as inhibitors of the mitochondrial pyruvate carrier (MPC) [41, 42]. The MPC plays a vital role in the coupling of glycolysis and mitochondrial respiratory processes by shuttling cytosolic pyruvate into the mitochondria where it can be utilized in the TCA cycle and OxPhos [43]. It is quite possible that the ability of compounds 3 and 9 to disrupt mitochondrial respiration may in part be due to inhibition of mitochondrial pyruvate uptake through interaction with the MPC.

Although MST results indicated significant inhibition in mitochondrial activity, fluorescence studies using both compound 9 and MitoTracker CMXROS did not reveal obvious co-localization of 9 in mitochondria (Figure 4). Cells exposed to 9 did not exhibit significant decrease in mitochondrial membrane potential in the time frames tested but rather, an apparent and acute hyperpolarization of the mitochondria. This observation was surprising as we had observed large amounts of proton leak in MST (Figures 2F and 3F) and may be due to a lack of glucose in media during microscopy experiments. Most of the compound 9 fluorescence appeared to reside in vesicular structures, which raises the possibility that it is being concentrated in endosomes, or lysosomes. It is currently unknown how the fluorescence characteristics of 9 are affected by distinct microenvironments associated with different organelles and cellular locations (e.g., pH or membrane polarization status). Hence, the fluorescence observed in these experiments may not represent the full extent of its actual intracellular distribution. Nevertheless, it is apparent that it enters both MDA-MB-231 and WiDr cells to readily detectable levels, and so a difference in cell entry does not appear to be the primary mechanism underlying the different sensitivities of these two lines to 9.

We then investigated the efficacy of candidate compounds 3 and 9 in WiDr mouse xenograft models. Treatment with compounds 3 and 9 in mice for three weeks provided equal efficacy with 35% and 33% tumor growth reduction, respectively [32, 33] (Figure 6A). Encouraged by these *in vivo* results, we further advanced 9 for efficacy studies in MDA-MB-231 xenograft model as a single agent and also in combination with a clinical breast cancer drug doxorubicin. Compound 9 showed significant tumor growth inhibition in both the cases (Figure 6B and 6C). We attribute the anticancer efficacy properties of dual MCT1 and MCT4 inhibitor 9 to a combination of direct or indirect effects resulting in metabolic disruption via inhibition of glycolysis and mitochondrial respiration (Figure 6D and 6E), along with cell cycle disruption (Supplementary Figure 6).

In conclusion, we developed 2-alkoxy-*N,N*-dialkyl cyanocinnamates 1–9 as potent and dual MCT1 and MCT4 inhibitors with activities at low nM concentrations. We carried out *in vitro* cell proliferation inhibition studies of these inhibitors in MCT1 and MCT4 expressing cancer cells and identified compound 9 as a lead candidate for further studies. Homology modeling and molecular docking prediction of compound 9 indicated that phenyl rings were involved in hydrophobic interactions and polar functional groups formed several putative hydrogen bonds with amino acid restudies of MCT1 and MCT4. Compounds 3 and 9 were evaluated for their glycolysis and mitochondrial OxPhos inhibition properties using extracellular flux assays. These compounds showed significant inhibition of glycolytic capacity, glycolytic reserve, maximal respiration, and spare respiratory capacity in MCT1 expressing WiDr and MCT4 expressing MDA-MB-231 cells. Compound 9 was found to be superior to 3 in inhibiting glycolytic and mitochondrial parameters in both cell lines. Florescence microscopy studies provided further proof that 9 was internalized and concentrated in areas near mitochondria in MDA-MB-231 and WiDr cells. Compound 3 was evaluated for its *in vivo* efficacy in WiDr tumor model in mice and compared it with 9 and this study indicated that both these inhibitors exhibited similar anticancer efficacy. Compound 9 was further advanced for *in vivo* study in MDA-MB-231 tumor xenograft models in mice and these results indicated that 9 significantly inhibited tumor growth as a single agent. These findings constitute the first report on the discovery of dual and potent MCT1 and MCT4 inhibitors with significant mitochondrial OxPhos inhibition properties. Owing to the importance of MCTs in tumor metabolism in several cancers, we believe that these inhibitors have good potential to be developed as broad-spectrum anticancer agents.

## MATERIALS AND METHODS

### Cell lines and culture conditions

MDA-MB-231 cells (ATCC, 2015) were grown in DMEM supplemented with 10% FBS and penicillin-streptomycin (50 U/ml, 50 μg/ml, Invitrogen). WiDr cells (ATCC, 2017) were cultured in MEM medium supplemented with 10% FBS (Atlanta Biologicals) and penicillin-streptomycin (50 U/ml, 50 μg/ml). For *in vitro* experiments, after seeding, cells were incubated at 37°C in 5% CO_2_ for 18–24 hours before the addition of test compounds.

### MCT4 inhibition assay

In this study, an L-[^14^C]-lactate based transport assay was developed by us to quantify MCT4 transport and its inhibition by test compounds. Previously, for MCT1 transport study, the pH of HEPES buffer with L-[^14^C]-lactate was maintained at 7.43 and lactate influx was quantified under this pH gradient condition. 2 × 10^5^ cells/mL were used for the MCT1 assay and the plates were incubated for 20 minutes after the addition of test compounds [32, 33]. For the MCT4 transport assay, the pH of HEPES buffer with L-[^14^C]-lactate was adjusted to 7.0 such that lactate influx into the cells was aided by the pH gradient. 4 × 10^5^ MDA-MB-231 cells/mL and incubation with test compounds for one hour was found to be optimal for isotope readings for this study. Test compounds were diluted to working concentration in HEPES buffer (140 mM NaCl, 5 mM KCl, 2 mM CaCl_2_, 2 mM MgCl_2_, 10 mM HEPES, pH 7.0) containing 3 μM L-[^14^C]-lactate (Perkin Elmer) and 2 μM L-lactate. Cells (24-well plate) were washed twice with 500 μL HEPES buffer and allowed to equilibrate for 15–20 minutes at 37°C. HEPES buffer was replaced with 250 μL test solution. After 1 hour, media was replaced with 500 μL ice-cold stop buffer (0.1 mM CHC solution in HBS, pH 7.4) and the plates were placed on ice. Cells were washed twice with ice-cold stop buffer and solubilized using 250 μL of 0.1 M NaOH in 5% Triton-X (Millipore Sigma). A 150 μL aliquot from each well was added to 4 mL EcoLite(+)^™^ scintillation fluid (MP Biomedicals) and radioactivity was determined by scintillation spectrometry. Inhibition by each test solution was calculated as a percentage of the maximum control uptake. CHC and dimethylsulfoxide (DMSO) were used as controls.

### Sulforhodamine-B (SRB) cell proliferation inhibition assay

Cells (5 × 10^4^ cells/mL) were cultured in 48-well plates. Test compounds were dissolved in DMSO (final concentration of DMSO is <0.1%) and were added to culture wells at various concentrations in replicate and incubated for 72 hours. Growth medium was removed and the wells were washed with PBS and dried. SRB (0.5% in 1% acetic acid) was added to the wells and incubated for 30–45 minutes. The wells were washed 3 times with 1% acetic acid and dried. The cellular protein was dissolved in trizma base (10 mM, pH 10.2) and absorbance was recorded at 540 nm. Percent survival was calculated using the formula $\%\text{Survival}=\frac{{\text{Abs}}_{\text{test compound}}}{{\text{Abs}}_{\text{control}}}\times 100$.

### Seahorse XFe96^®^ assessment of glycolysis and mitochondrial respiration

Extracellular acidification rates (ECAR) and oxygen consumption rates (OCR) were recorded in real-time for glycolysis stress test (GST) and mitochondrial stress test (MST), respectively, using Agilent Seahorse XFe96^®^ analyzer [44, 45].

### Fluorescent microscopy studies

MDA-MB-231 or WiDr cells (5 × 10^4^ cells/mL) were seeded in MatTek glass-bottom dishes (MatTek Corp, #P35G010C) and incubated for 48 hours and exposed to compound 9 (30 μM) for 1 h. MitoTracker Red CMXROS (Invitrogen, M7512, 100 nM) was added 15 minutes prior to imaging. Media was then aspirated and replaced with PBS + 5% FBS for imaging. Cells were imaged using a Nikon TE2000 epifluorescent microscope and a Photometrics Dyno CCD camera.

### Homology modeling of and molecular docking to human MCT1 and MCT4 structures

Structures were generated for human MCT1 and MCT4 by homology modeling with MODELLER 9.18 using inward-open human glucose transporter 1 as a structural template, PDB file: 5eqi [46, 47]. Due to minimal sequence similarity, we generated a final template alignment by consensus sequence alignment guided by consensus transmembrane spanning domain prediction followed by manual adjustment to eliminate gaps in the putative transmembrane spanning domains. The last 50 C-terminal amino acids were deleted but are not part of a transmembrane spanning domain. As with the homology model of rat MCT1 previously built by Manoharan, et. al., we consider the models synthesized to be of intermediate quality but predictive in nature [38]. Autodock Vina was used to dock parent compound CHC and compound 9 to the inward open homology models [48]. From estimated individual binding energies, a crude difference between CHC and compound 9 affinity was calculated. Further, the number of poses nearly identical to the most favorable docked pose was used as a surrogate for binding specificity.

### Ethics statement

The animal studies were approved and conducted by GenScript Corporation (Piscataway, NJ, USA) according to their approved IACUC protocols.

### Tumor growth inhibition studies

Tumor cells suspended in 1:1 matrigel-PBS were injected on right flank of female SCID mice (*n* = 6 mice/group, 10^7^ MDA-MB-231 cells) or right flank of female athymic nude mice (*n* = 8 mice/group, 5 × 10^6^ WiDr cells). Tumors were measured using calipers every 2–3 days and tumor volumes were calculated using the formula V = ab^2^/2 where ‘a’ is the long diameter of the tumor and ‘b’ is the short diameter of the tumor. Tumor growth inhibition was determined using the formula % inhibition = [(C – T)/C] × 100 where C is average tumor weight of the control group and T is the average tumor weight of the test group.

### Statistical analysis

Statistics were computed using GraphPad Prism 6.0. For *in vitro* studies, repeated measures one-way ANOVA and for *in vivo* studies, Mann-Whitney test were used to compare the treated and untreated groups. A *P*-value of < 0.05 was considered significant.

## SUPPLEMENTARY MATERIALS FIGURES AND TABLES



## Abbreviations

CHC: α-cyano-4-hydroxy cinnamic acid
MCT: monocarboxylate transporter
OxPhos: oxidative phosphorylation
SRB: sulforhodamine-B
DMSO: dimethylsulfoxide
ECAR: extracellular acidification rate
OCR: oxygen consumption rate
GST: glycolysis stress test
MST: mitochondrial stress test
RBE4: rat brain endothelial-4
TNBC: triple negative breast cancer
MPC: mitochondrial pyruvate carrier

## References

[1] Fritz V, Fajas L (2010). Metabolism and proliferation share common regulatory pathways in cancer cells. Oncogene.

[2] Hanahan D, Weinberg RA (2011). Hallmarks of cancer: the next generation. Cell.

[3] Cantor JR, Sabatini DM (2012). Cancer cell metabolism: one hallmark, many faces. Cancer Discov.

[4] Pecqueur C, Oliver L, Oizel K, Lalier L, Vallette FM (2013). Targeting metabolism to induce cell death in cancer cells and cancer stem cells. Int J Cell Biol.

[5] Pavlova NN, Thompson CB (2016). The Emerging Hallmarks of Cancer Metabolism. Cell Metab.

[6] Lee N, Kim D (2016). Cancer Metabolism: Fueling More than Just Growth. Mol Cells.

[7] DeBerardinis RJ, Chandel NS (2016). Fundamentals of cancer metabolism. Sci Adv.

[8] Kalyanaraman B (2017). Teaching the basics of cancer metabolism: developing antitumor strategies by exploiting the differences between normal and cancer cell metabolism. Redox Biol.

[9] Martinez-Outschoorn UE, Balliet RM, Rivadeneira DB, Chiavarina B, Pavlides S, Wang C, Whitaker-Menezes D, Daumer KM, Lin Z, Witkiewicz AK, Flomenberg N, Howell A, Pestell RG (2010). Oxidative stress in cancer associated fibroblasts drives tumor-stroma co-evolution: A new paradigm for understanding tumor metabolism, the field effect and genomic instability in cancer cells. Cell Cycle.

[10] Pavlides S, Vera I, Gandara R, Sneddon S, Pestell RG, Mercier I, Martinez-Outschoorn UE, Whitaker-Menezes D, Howell A, Sotgia F, Lisanti MP (2012). Warburg meets autophagy: cancer-associated fibroblasts accelerate tumor growth and metastasis via oxidative stress, mitophagy, and aerobic glycolysis. Antioxid Redox Signal.

[11] Martinez-Outschoorn UE, Lin Z, Whitaker-Menezes D, Howell A, Lisanti MP, Sotgia F (2012). Ketone bodies and two-compartment tumor metabolism: stromal ketone production fuels mitochondrial biogenesis in epithelial cancer cells. Cell Cycle.

[12] Lee M, Yoon JH (2015). Metabolic interplay between glycolysis and mitochondrial oxidation: the reverse Warburg effect and its therapeutic implication. World J Biol Chem.

[13] Fu Y, Liu S, Yin S, Niu W, Xiong W, Tan M, Li G, Zhou M (2017). The reverse Warburg effect is likely to be an Achilles' heel of cancer that can be exploited for cancer therapy. Oncotarget.

[14] Ganapathy V, Thangaraju M, Prasad PD (2009). Nutrient transporters in cancer: relevance to Warburg hypothesis and beyond. Pharmacol Ther.

[15] Ganapathy-Kanniappan S, Geschwind JF (2013). Tumor glycolysis as a target for cancer therapy: progress and prospects. Mol Cancer.

[16] Liberti MV, Locasale JW (2016). The Warburg Effect: How Does it Benefit Cancer Cells?. Trends Biochem Sci.

[17] Jiang B (2017). Aerobic glycolysis and high level of lactate in cancer metabolism and microenvironment. Genes Dis.

[18] Granchi C, Fancelli D, Minutolo F (2014). An update on therapeutic opportunities offered by cancer glycolytic metabolism. Bioorg Med Chem Lett.

[19] Roy D, Sheng GY, Herve S, Carvalho E, Mahanty A, Yuan S, Sun L (2017). Interplay between cancer cell cycle and metabolism: Challenges, targets and therapeutic opportunities. Biomed Pharmacother.

[20] Marchetti P, Guerreschi P, Mortier L, Kluza J (2015). Integration of Mitochondrial Targeting for Molecular Cancer Therapeutics. Int J Cell Biol.

[21] Weinberg SE, Chandel NS (2015). Targeting mitochondria metabolism for cancer therapy. Nat Chem Biol.

[22] Kim HK, Noh YH, Nilius B, Ko KS, Rhee BD, Kim N, Han J (2017). Current and upcoming mitochondrial targets for cancer therapy. Semin Cancer Biol.

[23] Halestrap AP (2013). The SLC16 gene family - structure, role and regulation in health and disease. Mol Aspects Med.

[24] Baltazar F, Pinheiro C, Morais-Santos F, Azevedo-Silva J, Queirós O, Preto A, Casal M (2014). Monocarboxylate transporters as targets and mediators in cancer therapy response. Histol Histopathol.

[25] Jones RS, Morris ME (2016). Monocarboxylate Transporters: Therapeutic Targets and Prognostic Factors in Disease. Clin Pharmacol Ther.

[26] Ruan Y, Zeng F, Cheng Z, Zhao X, Fu P, Chen H (2017). High expression of monocarboxylate transporter 4 predicts poor prognosis in patients with lung adenocarcinoma. Oncol Lett.

[27] Latif A, Chadwick AL, Kitson SJ, Gregson HJ, Sivalingam VN, Bolton J, McVey RJ, Roberts SA, Marshall KM, Williams KJ, Stratford IJ, Crosbie EJ (2017). Monocarboxylate Transporter 1 (MCT1) is an independent prognostic biomarker in endometrial cancer. BMC Clin Pathol.

[28] Payen VL, Hsu MY, Rädecke KS, Wyart E, Vazeille T, Bouzin C, Porporato PE, Sonveaux P (2017). Monocarboxylate transporter MCT1 promotes tumor metastasis independently of its activity as a lactate transporter. Cancer Res.

[29] Johnson JM, Cotzia P, Fratamico R, Mikkilineni L, Chen J, Colombo D, Mollaee M, Whitaker-Menezes D, Domingo-Vidal M, Lin Z, Zhan T, Tuluc M, Palazzo J (2017). MCT1 in Invasive Ductal Carcinoma: Monocarboxylate Metabolism and Aggressive Breast Cancer. Front Cell Dev Biol.

[30] Critchlow SE, Tate L (2010). Use of a MCT1 inhibitor in the treatment of cancers expressing MCT1 over MCT4. https://patents.google.com/patent/WO2010089580A1/en.

[31] Draoui N, Schicke O, Seront E, Bouzin C, Sonveaux P, Riant O, Feron O (2014). Antitumor activity of 7-aminocarboxycoumarin derivatives, a new class of potent inhibitors of lactate influx but not efflux. Mol Cancer Ther.

[32] Gurrapu S, Jonnalagadda SK, Alam MA, Nelson GL, Sneve MG, Drewes LR, Mereddy VR (2015). Monocarboxylate transporter 1 inhibitors as potential anticancer agents. ACS Med Chem Lett.

[33] Mereddy VR, Drewes LR, Alam MA, Jonnalagadda SK, Gurrapu S (2016). Therapeutic Compounds. https://app.dimensions.ai/details/patent/US-9296728-B2.

[34] Gurrapu S, Jonnalagadda SK, Alam MA, Ronayne CT, Nelson GL, Solano LN, Lueth EA, Drewes LR, Mereddy VR (2016). Coumarin carboxylic acids as monocarboxylate transporter 1 inhibitors: in vitro and in vivo studies as potential anticancer agents. Bioorg Med Chem Lett.

[35] Parnell KM, McCall J (2016). MCT4 Inhibitors for Treating Disease. https://patents.google.com/patent/US20160362378A1/en.

[36] Curtis NJ, Mooney L, Hopcroft L, Michopoulos F, Whalley N, Zhong H, Murray C, Logie A, Revill M, Byth KF, Benjamin AD, Firth MA, Green S (2017). Pre-clinical pharmacology of AZD3965, a selective inhibitor of MCT1: DLBCL, NHL and Burkitt's lymphoma anti-tumor activity. Oncotarget.

[37] Noble RA, Bell N, Blair H, Sikka A, Thomas H, Phillips N, Nakjang S, Miwa S, Crossland R, Rand V, Televantou D, Long A, Keun HC (2017). Inhibition of monocarboxyate transporter 1 by AZD3965 as a novel therapeutic approach for diffuse large B-cell lymphoma and Burkitt lymphoma. Haematologica.

[38] Manoharan C, Wilson MC, Sessions RB, Halestrap AP (2006). The role of charged residues in the transmembrane helices of monocarboxylate transporter 1 and its ancillary protein basigin in determining plasma membrane expression and catalytic activity. Mol Membr Biol.

[39] Nancolas B, Sessions RB, Halestrap AP (2015). Identification of key binding site residues of MCT1 for AR-C155858 reveals the molecular basis of its isoform selectivity. Biochem J.

[40] Juel C, Halestrap AP (1999). Lactate transport in skeletal muscle - role and regulation of the monocarboxylate transporter. J Physiol.

[41] Divakaruni AS, Wallace M, Buren C, Martyniuk K, Andreyev AY, Li E, Fields JA, Cordes T, Reynolds IJ, Bloodgood BL, Raymond LA, Murphy AN (2017). Inhibition of the mitochondrial pyruvate carrier protects from excitotoxic neuronal death. J Cell Biol.

[42] Divakaruni AS, Wiley SE, Rogers GW, Andreyev AY, Petrosyan S, Loviscach M, Wall EA, Yadava N, Heuck AP, Ferrick DA, Henry RR, McDonald WG, Colca JR (2013). Thiazolidinediones are acute, specific inhibitors of the mitochondrial pyruvate carrier. Proc Natl Acad Sci USA.

[43] Vacanti NM, Divakaruni AS, Green CR, Parker SJ, Henry RR, Ciaraldi TP, Murphy AN (2014). Regulation of substrate utilization by the mitochondrial pyruvate carrier. Mol Cell.

[44] TeSlaa T, Teitell MA (2014). Techniques to monitor glycolysis. Methods Enzymol.

[45] Jitschin R, Hofmann AD, Bruns H, Giessl A, Bricks J, Berger J, Saul D, Eckart MJ, Mackensen A, Mougiakakos D (2014). Mitochondrial metabolism contributes to oxidative stress and reveals therapeutic targets in chronic lymphocytic leukemia. Blood.

[46] Šali A, Blundell TL (1993). Comparative protein modelling by satisfaction of spatial restraints. J Mol Biol.

[47] Kapoor K, Finer-Moore JS, Pedersen BP, Caboni L, Waight A, Hillig RC, Bringmann P, Heisler I, Müller T, Siebeneicher H, Stroud RM (2016). Mechanism of inhibition of human glucose transporter GLUT1 is conserved between cytochalasin B and phenylalanine amides. Proc Natl Acad Sci USA.

[48] Trott O, Olson AJ (2010). AutoDock Vina: improving the speed and accuracy of docking with a new scoring function, efficient optimization, and multithreading. J Comput Chem.

