# Supplementary material for: Novel N,N-dialkyl cyanocinnamic acids as monocarboxylate transporter 1 and 4 inhibitors
Source: Oncotarget. 2019 Mar 22;10(24):2355–68. doi: 10.18632/oncotarget.26760 (PMC6481325; doi:10.18632/oncotarget.26760)
Supplement: Supplementary file 1 [file oncotarget-10-2355-s001.pdf]

Novel N,N-dialkyl cyanocinnamic acids as monocarboxylate transporter 1 and 4 inhibitors

SUPPLEMENTARY MATERIALS

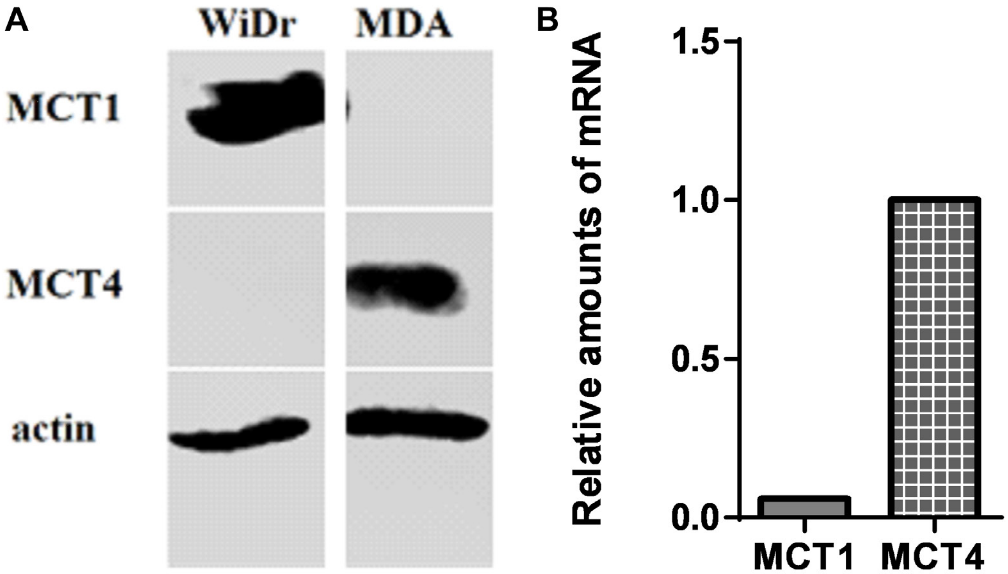

Supplementary Figure 1: (**A**) Western blot analysis of MCT1 and MCT4 expression in colorectal adenocarcinoma cell line WiDr and triple negative breast cancer cell line MDA-MB-231 indicate that WiDr expresses high MCT1 and undetectable levels of MCT4 whereas MDA-MB-231 exhibits high MCT4, but undetectable levels of MCT1 expression; MCT1 (rabbit polyclonal IgG MCT1, Origene, TA321555) and MCT4 (rabbit polyclonal IgG MCT4, Santa Cruz, sc50329) (**B**) Quantitative PCR analysis of MDA-MB-231 cell line shows low MCT1 expression (~6%) and high MCT4 expression. Primers for MCT1: forward-GCTGCTTCTGTTGTTGCGAATGGA, reverse-AAAGGCAAATCCAAAGACTCCCGC; for MCT4: forward-CTCTGCCTAAGGGACAAAGAAA, reverse-AGAACCACACAGCTCCTAGA. All the primers used here were designed through the PrimerQuest tool from Integrated DNA Technologies.

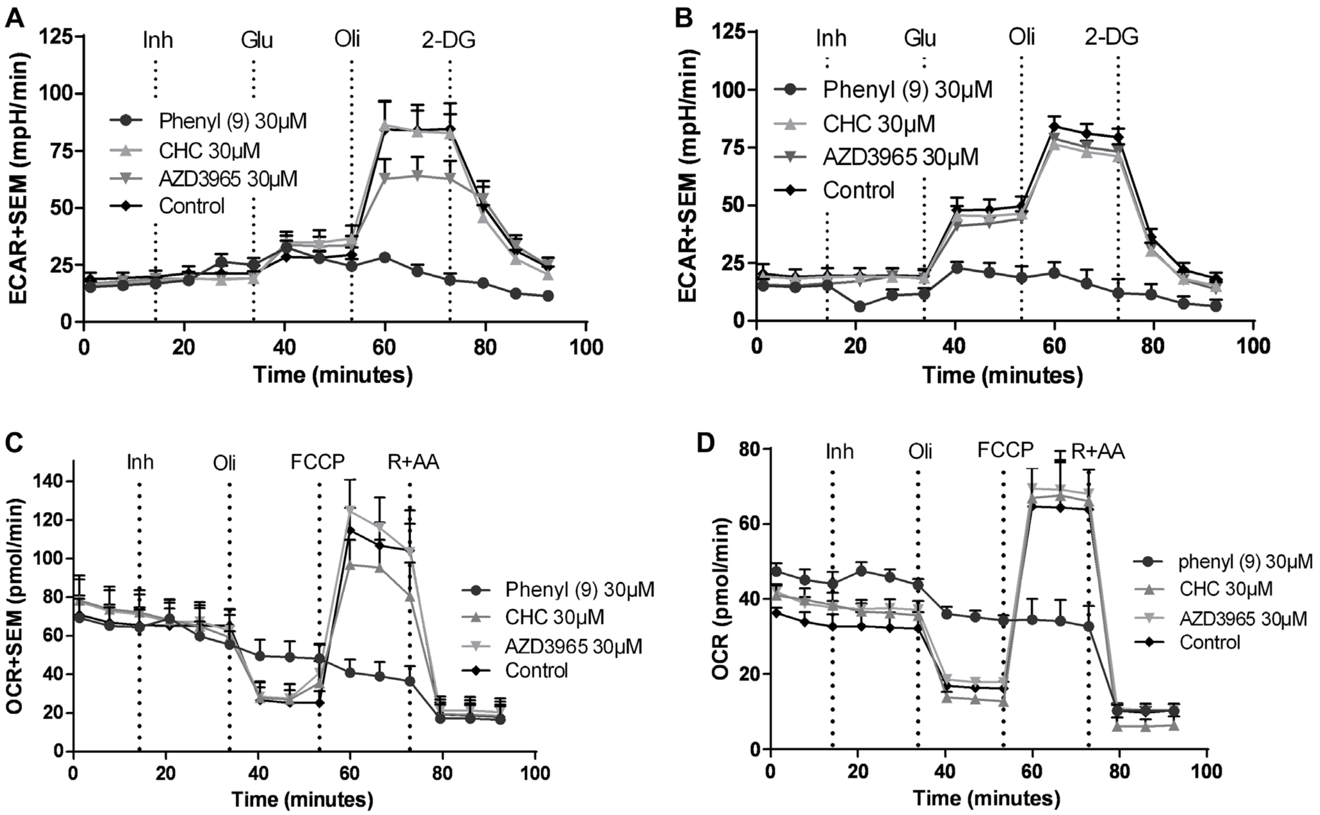

Supplementary Figure 2: Glycolysis stress test of compounds phenyl 9, CHC, and AZD3965 at 30 µM concentration in (**A**) MCT1 expressing WiDr, and (**B**) MCT4 expressing MDA-MB-231 cells. Mitochondrial stress test of compounds phenyl 9, CHC, and AZD3965 at 30 µM concentration in (**C**) MCT1 expressing WiDr, and (**D**) MCT4 expressing MDA-MB-231 cells.

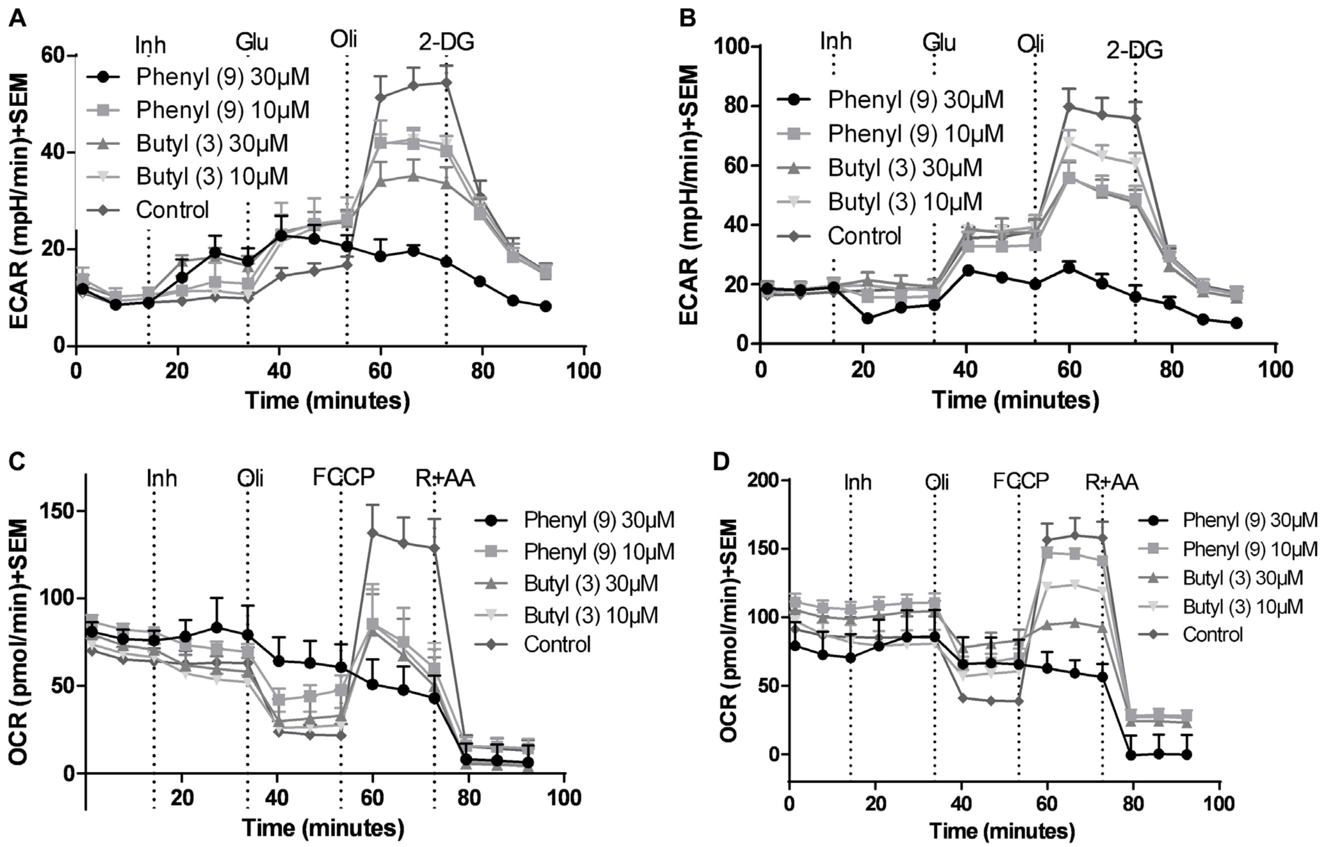

Supplementary Figure 3: Glycolysis stress test of compounds butyl 3 and phenyl 9 at 30 μM concentration in (**A**) MCT1 expressing WiDr, and (**B**) MCT4 expressing MDA-MB-231 cells. Mitochondrial stress test of compounds phenyl 9 and butyl 3 at 10 and 30 μM concentration in (**C**) MCT1 expressing WiDr, and (**D**) MCT4 expressing MDA-MB-231 cells.

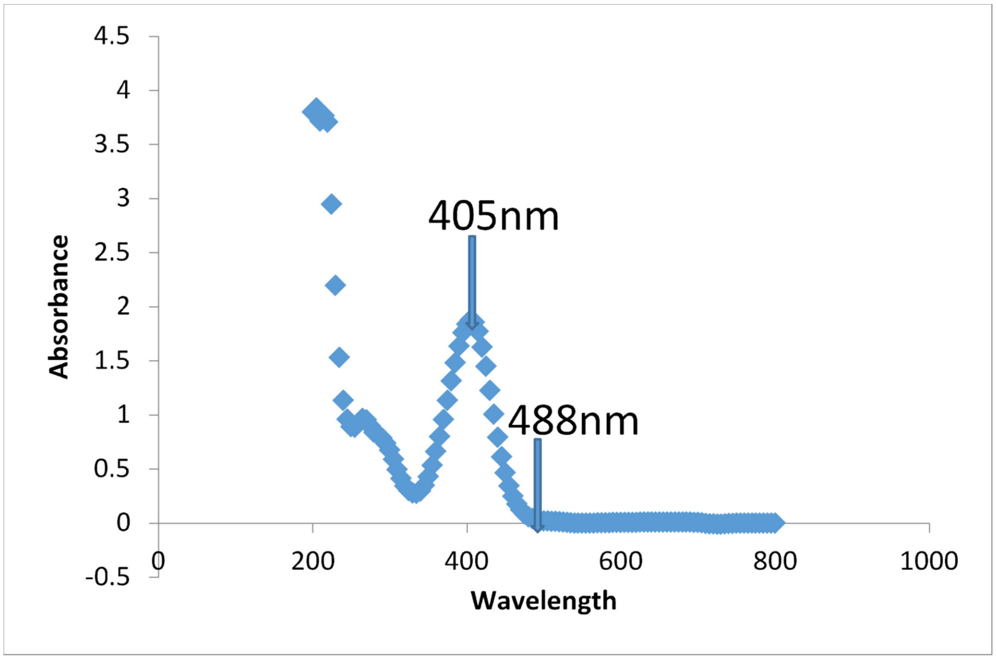

Supplementary Figure 4: Absorbance spectrum of compound 9 (200 μM). Note absorbance maximum at 405 nm. For *in vitro* imaging, a lower energy wavelength is preferred for imaging to minimize cellular damage and autofluorescence. Note compound 9 absorbs light at 488 nm (FITC) excitation wavelength (488 nm). Spectrum was recorded using a Cary WinUV 60 spectrophotometer (Agilent).

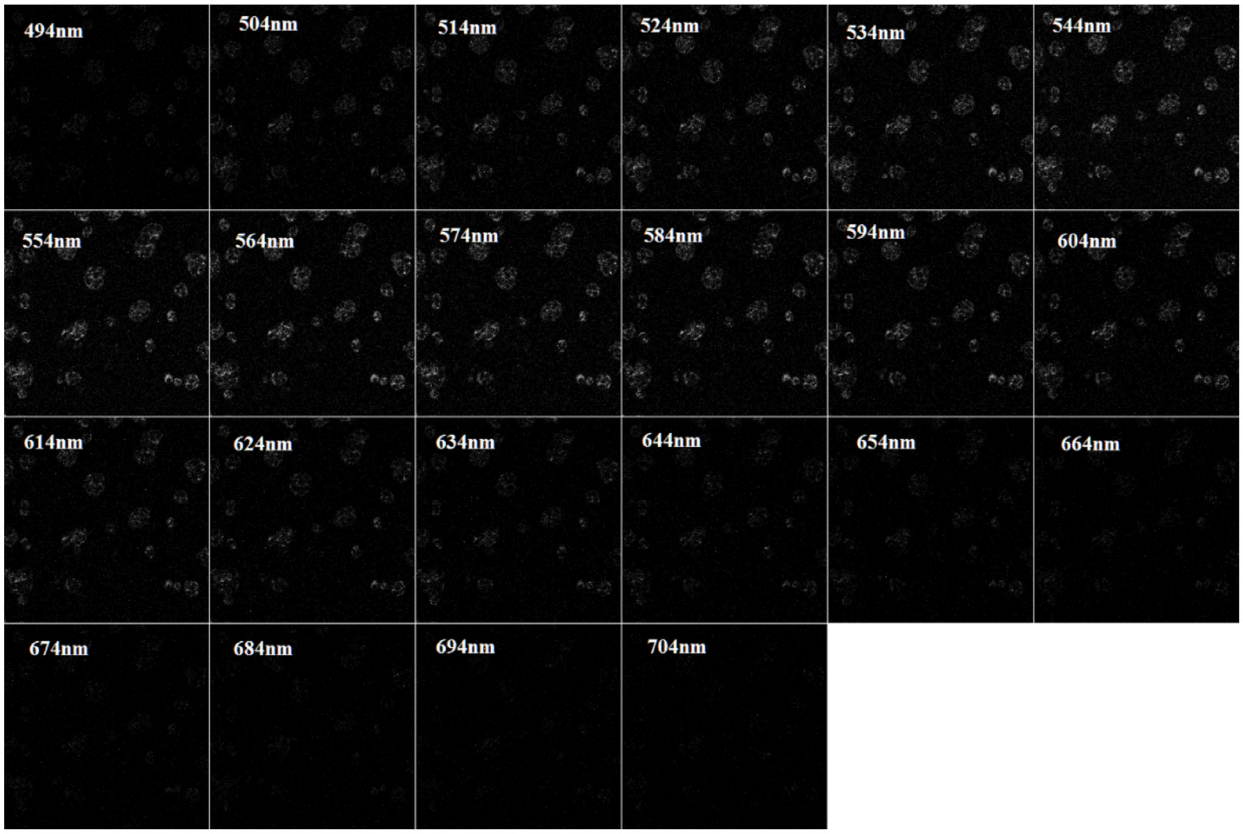

Supplementary Figure 5: Emission spectrum of compound 9 in MDA-MB-231 cells excited at 488 nm. Note compound fluorescence in 525 nm emission filter. Cells were plated in glass bottom MatTek dishes at 5 $\times 10^4$ cells/ml and incubated for 48 hours. Cells were then exposed to compound 9 at 30 μM for one hour, rinsed with PBS, and imaged in PBS + 5% FBS. Images were captured using a 20x phase contrast lens using a Zeiss LSM710 confocal laser scanning microscope.

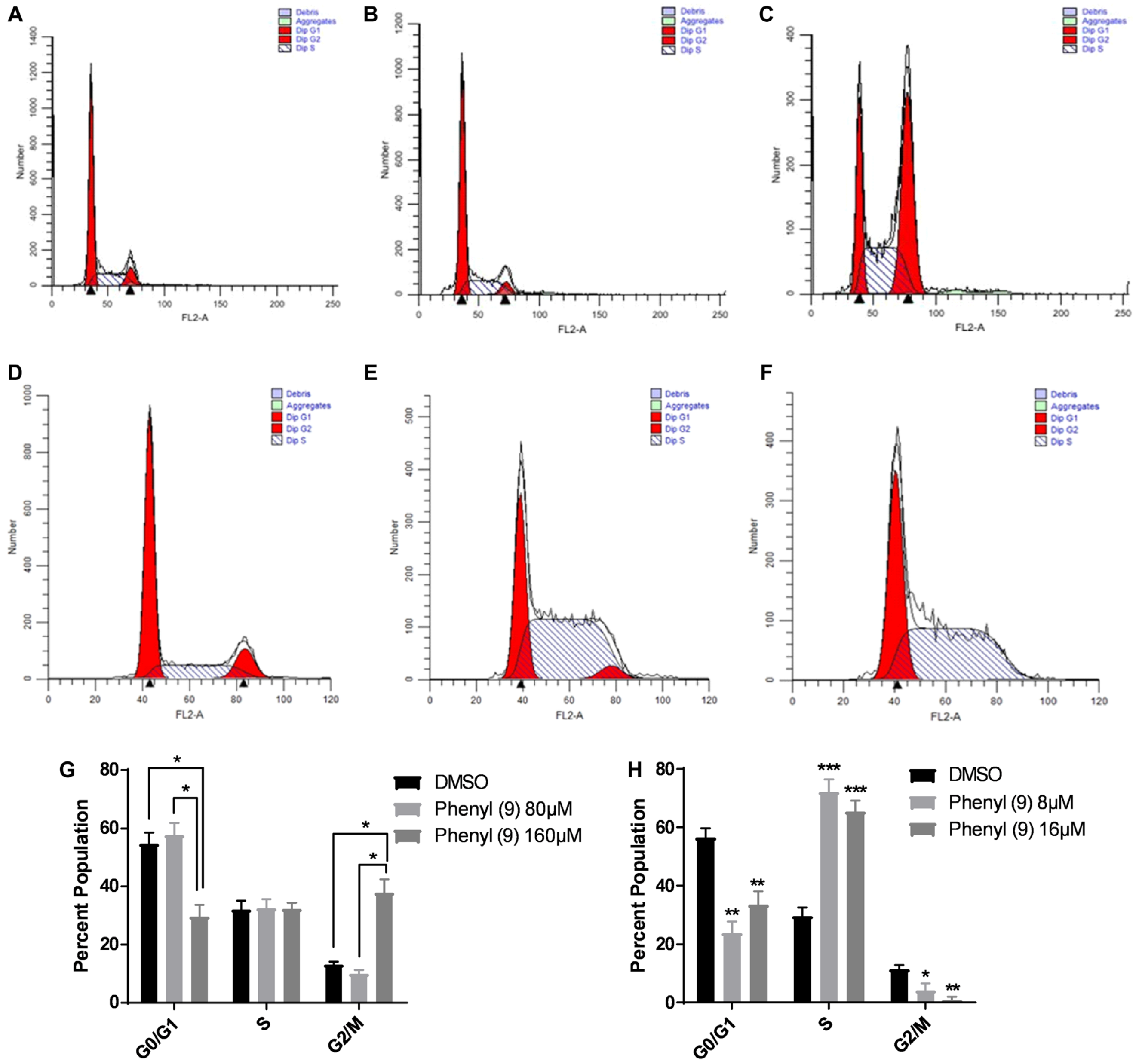

Supplementary Figure 6: Effect of compound 9 on cell-cycle distributions in MDA-MB-231 and WiDr cells. Representative examples of FCM derived histograms of MDA-MB-231 cells treated with (**A**) DMSO, (**B**) 80 μM 9, (**C**) 160 μM 9 and WiDr cells with (**D**) DMSO, (**E**) 8 μM 9, and (**F**) 16 μM 9. Cell-cycle distributions were calculated using ModFitLT 5.0 software. Doublet-discrimination was performed prior to ModFit analysis utilizing FL2-area and FL2-width dot plots to control for cell clumping during enumeration of DNA content. Average + sem of G_0/G_1, S and G_2/M populations of minimum three individual experiments in (**G**) MDA-MB-231 and (**H**) WiDr cell lines were calculated and plotted. Statistical significance was calculated using repeated measures one-way ANOVA ($^*P < 0.05$). DMSO treated cells were used as controls.

Supplementary Table 1: Lactate uptake assay for MCT1 and MCT4 IC_{50} (nM) values of 2-methoxy *N*,*N*-dialkyl cyanocinnamates 1–9

Compound	MCT1 IC_{50}	MCT4 IC_{50}
Propyl (1)	12 ± 1	11 ± 1
Allyl (2)	29 ± 2	24 ± 6
Butyl (3)	9 ± 1	14 ± 2
Isobutyl (4)	11 ± 4	17 ± 2
Pentyl (5)	34 ± 7	85 ± 8
Pyrrolidinyl (6)	48 ± 20	48 ± 4
Piperidinyl (7)	25 ± 5	58 ± 13
Benzyl (8)	25 ± 3	32 ± 3
Phenyl (9)	8 ± 1	18 ± 8
Na salt of 9	11 ± 3	23 ± 1

These values were determined using MCT1 expressing RBE4 and MCT4 expressing MDA-MB-231 cell lines. Average ± SEM of minimum three separate experiments.

Supplementary Table 2: Residues within 4.5A of inhibitor phenyl 9 in the best ranked docking poses of MCT1 and MCT4

MCT 1		MCT4	MCT 1		MCT4
Tyr 34	---	Tyr 36	Phe 363		
Leu 128	---	Phe 130			Ser 331
		Leu 134			Tyr 332
		Asn 149	Leu 370	---	Val 335
Met 151			Ser 371	---	Gly 336
Ser 154	---	Ser 156	Ser 372	---	Ala 337
		Pro 157	Leu 374	---	Gln 339
Ile 272	---	Val 237	Phe 375	---	Phe 340
Leu 277	---	Leu 242	Leu 378		
Phe 278	---	Phe 243			Ile 356
Leu 281			Val 394	---	Val 359
Arg 313	---	Arg 278	Thr 395	---	Leu 360
			Glu 398	---	Glu 363

Adjacent residues are analogous amino acids in the aligned sequences.
